# The Effect of Comprehensive Medical Care on the Long-Term Outcomes of Children Discharged from the NICU with Tracheostomy

**DOI:** 10.2174/1874306401812010039

**Published:** 2018-07-31

**Authors:** Wilfredo De Jesus-Rojas, Ricardo A. Mosquera, Cheryl Samuels, Julie Eapen, Traci Gonzales, Tomika Harris, Sandra McKay, Fatima Boricha, Claudia Pedroza, Chiamaka Aneji, Amir Khan, Cindy Jon, Katrina McBeth, James Stark, Aravind Yadav, Jon E. Tyson

**Affiliations:** 1 Division of Pulmonary Medicine/Allergy & Immunology/Rheumatology, Department of Pediatrics, McGovern Medical School, University of Texas Health Science Center, Houston, TX, USA; 2 High-Risk Children’s Clinic, McGovern Medical School, University of Texas Health Science Center, Houston, TX, USA; 3 Division of Neonatal-Perinatal Medicine, Department of Pediatrics, McGovern Medical School, University of Texas Health Science Center, Houston, TX, USA

**Keywords:** Decannulation, Tracheostomy, Complex-care, Mortality, Comprehensive-care, Usual-care

## Abstract

**Background:**

Survival of infants with complex care has led to a growing population of technology-dependent children. Medical technology introduces additional complexity to patient care. Outcomes after NICU discharge comparing Usual Care (UC) with Comprehensive Care (CC) remain elusive.

**Objective:**

To compare the outcomes of technology-dependent infants discharged from NICU with tracheostomy following UC versus CC.

**Methods:**

A single site retrospective study evaluated forty-three (N=43) technology-dependent infants discharged from NICU with tracheostomy over 5½ years (2011-2017). CC provided 24-hour accessible healthcare-providers using an enhanced medical home. Mortality, total hospital admissions, 30-days readmission rate, time-to-mechanical ventilation liberation, and time-to-decannulation were compared between groups.

**Results:**

CC group showed significantly lower mortality (3.4%) versus UC (35.7%), RR, 0.09 [95%CI, 0.12-0.75], P=0.025. CC reduced total hospital admissions to 78 per 100 child-years versus 162 for UC; RR, 0.48 [95% CI, 0.25-0.93], P=0.03. The 30-day readmission rate was 21% compared to 36% in UC; RR, 0.58 [95% CI, 0.21-1.58], P=0.29). In competing-risk regression analysis (treating death as a competing-risk), hazard of having mechanical ventilation removal in CC was two times higher than UC; SHR, 2.19 [95% CI, 0.70-6.84]. There was no difference in time-to-decannulation between groups; SHR, 1.09 [95% CI, 0.37-3.15].

**Conclusion:**

CC significantly decreased mortality, total number of hospital admissions and length of time-to-mechanical ventilation liberation.

## 1 INTRODUCTION

Although survival of technology-dependent infants has increased [1], the outcomes after discharge from the Neonatal Intensive Care Unit (NICU) remain uncertain. Current advancements in obstetrical and neonatal care have shown a marked increase in survival rate for infants admitted to the NICU over the past few decades [2]. At the same time, the survival of infants requiring a complex medical care has led to a growing population of technology-dependent infants with significant medical needs [3, 4]. Technology-dependent infants require a higher level of care, intensive parental teaching and discharge coordination from a multidisciplinary team both in the hospital and at home [5]. The process required for a technology-dependent infant to experience a successful home transition and potentially achieve a non-technology dependent state, can prove to be challenging [6]. Adhering to a child’s medical care plan can be difficult in a complex and fragmented health care system [7-10]. The use of comprehensive care provided in an enhanced medical home can mitigate the patient and caretaker difficulties associated with the care of a technology-dependent infant [4].

Our previous study demonstrated that providing care to children with chronic illnesses in a patient-centered enhanced medical home decreased both serious complications and cost [11]. Whether this comprehensive care model can produce similar benefits for technology-dependent infants after NICU discharge has not been previously evaluated. In contrast of a Usual Care model (UC), a Comprehensive Care model (CC) involves subspecialist, pediatricians and nurse practitioners, functioning as primary care providers delivering acute and chronic care along with care coordination between visits for a small template of patients. Patient visit lengths are extended, phone calls are made between visits, and family members are involved in the decision-making process. In addition, a multidisciplinary team is available to help support families of technology-dependent infants that include a social worker, nutritionist and subspecialists along with 24/7 telephone access to a healthcare provider for acute medical conditions. This model helps mitigate the many challenges, needs and barriers the families of children with medical complexity face on a regular basis [12].

In order to evaluate the impact of CC specifically on infants discharged from the NICU with tracheostomy, we conducted a single site retrospective study to compare outcomes of technology-dependent infants followed with UC versus CC. We described the baseline characteristics of technology-dependent infants after NICU discharge in both groups including: associated morbidity and mortality, timing to achieve liberation from mechanical ventilation and subsequent decannulation.

## 2 MATERIALS AND METHODS

A single site retrospective chart review was conducted with a cohort of forty-three (N=43) technology-dependent infants. All patients were discharged from Memorial Hermann Children’s Hospital NICU with a diagnosis of tracheostomy dependence. Enrollment occurred between January 2011 and December 2016, with outcome follow up through October 2017. Patients were followed in CC or UC with a pediatrician in the community and pediatric pulmonologist. The decision for an infant to follow UC versus CC was made jointly by family and healthcare team. Both groups were followed by outpatient pediatric Otorhinolaryngology subspecialist (ORL) for tracheostomy evaluation and management. We excluded patients with associated comorbidities that could further contribute to the development of chronic respiratory failure, including: severe Hypoxic-Ischemic-Encephalopathy (HIE), and Central Hypoventilation Syndrome (CCHS). Patients with Do Not Resuscitate (DNR) status, and Palliative Care status were also excluded. The University of Texas Health Science Center (UTH) institutional review board approved the study.

A review of the inpatient and outpatient electronic medical records of each patient was divided and conducted between all authors. Data obtained from the medical record included: gestational age, birth weight, race, gender, medical insurance, age at mechanical ventilation liberation and decannulation. Additional evaluation of patient comorbidities included: presence of BPD, oxygen dependence, gastrostomy or gastrojejunal tube, Nissen fundoplication, history of Intraventricular Hemorrhage (IVH), pulmonary hypertension diagnosed by echocardiogram (Right Ventricle Systolic Pressure [RVSP] above half systemic pressure), patent ductus arteriosus, Congenital Diaphragmatic Hernia (CDH), Tracheoesophageal Fistula (TEF), oromaxillofacial abnormalities, spinal muscular atrophy and Trisomy 21.

### 2.1 Usual Care

NICU follow up was provided by private pediatricians or at UTH pediatric clinics staffed by residents with pediatric faculty supervision. Availability for same day visits was not always offered. Patients were also treated at UTH subspecialty clinics with both pediatric ORL and pulmonary clinics for the management of respiratory diseases, pulmonary hypertension, mechanical ventilation liberation and tracheostomy care. Parent’s calls were taken by on-call faculty or supervised pediatric residents at nights or weekends. Emergency Department (ED) referrals may or may not be discussed with the ED staff.

### 2.2 Comprehensive Care

CC care was provided at UTH High-Risk Children’s Clinic (HRCC), a specialty clinic for children with chronic illnesses provided in an enhanced medical home model. A previous randomized controlled trial demonstrated that this level of care reduces ED visits and hospitalizations for children with medical complexity [13]. Pediatricians and pediatric nurse practitioners supervised by a pediatric pulmonologist, provided care for patients during the day and the same providers shared call with a cell phone 24-hours a day. A pediatric pulmonologist was available for consultation via phone as needed after hours. A Spanish-speaking provider or interpreter was available via phone, and each clinician could access medical records from home. In addition, during weekdays, a nutritionist and social worker were available. Monthly, specialists from pediatric gastroenterologist, infectious diseases, neurologist, and allergist/immunologist attended the clinic. Consultation with these subspecialists was promptly available when needed. Same day visits were scheduled for patients with acute illness presenting before 5:00 pm on weekdays. Night calls were managed over the telephone with a follow-up appointment the next workday when needed. If ED visits or hospitalizations were necessary, the HRCC provider contacted the ED clinicians to discuss the plan and provide additional management. After ED or hospitalization, a timely follow-up visit was arranged. Weekly meetings and patient discussions were completed to identify methods to improve patient care and reduce unnecessary ED visits [13] (Table **1**).

### 2.3 Statistics

Descriptive statistics are presented as percentages or medians with standard deviation. Differences in mortality and 30-day readmission rates were evaluated with Poisson Generalized Estimating Equation (GEE) models with robust standard errors to estimate Relative Risks (RR). Total hospital admissions were analyzed with a negative binomial regression model. Models for mortality and hospital admissions were adjusted for length of follow-up. Time to mechanical ventilation removal, and decannulation were analyzed with competing-risks survival regression models where death was treated as a competing event. A p-value < 0.05 was considered statistically significant. Data were analyzed using STATA 14 software (StataCorp. 2015. Stata Statistical Software: Release 14. College Station, TX: StataCorp LP).

## 3 RESULTS

### 3.1 Patient Characteristics

**Fig. (1):**
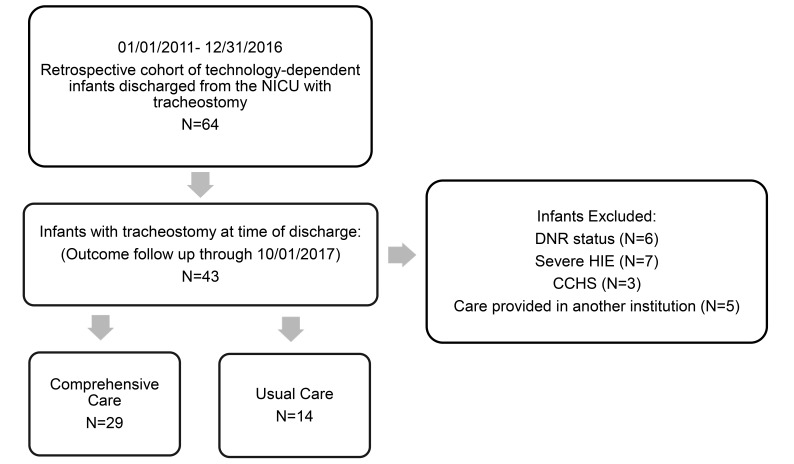
Diagram of inclusion and exclusion criteria. A total of 64 infants were enrolled. Patients with Do Not Resuscitate/Palliative Care status (DNR), Hypoxic-Ischemic Encephalopathy (HIE) and Central Congenital Hypoventilation Syndrome (CCHS) were excluded. After exclusion criteria, 43 technology-dependent infants meets inclusion criteria, 14 patients followed usual care and 29 comprehensive care over a period of 5½ years.

Between January 2011 and December 2016, technology-dependent infants discharged from the NICU with tracheostomy were enrolled (N=64). We excluded infants with DNR status (N=6) severe HIE (N=7), CCHS (N=3) and infants followed at another institution (N=5). The remaining 43 infants were followed until decannulation or the conclusion of the study (Fig. **1**).

There were a total of 41% females (P=0.36) and 37% Hispanics (P=0.22). Median age for a tracheostomy placement was 3.5 months (UC: 3 months versus CC: 4.3 months, P=0.14). The most common reasons for tracheostomy included: tracheobronchomalacia (67%) and subglottic stenosis (28%). A total of 86% of infants required long-term MV at the time of NICU discharge. Synchronized Intermittent Mandatory Ventilation (SIMV) on Pressure Controlled (PC) mode (41%) was the most commonly used ventilator support. Median age at NICU discharge did not differ among groups; UC was 5.8 months and CC was 6.5 months (P=0.55). Bronchopulmonary dysplasia (BPD) was present in 51% of our sample and 40% were oxygen dependent at discharge. A total of 69% of infants received a gastrostomy tube and 20% required a Nissen fundoplication, 14% of infants had IVH, and 11% required a ventriculoperitoneal shunt. 16% had Trisomy 21 and 14% were placed on anticonvulsive medications for seizures. Pulmonary hypertension was detected by echocardiogram (right ventricle systolic pressure [RVSP] above half systemic pressure) in 30% of patients. A total of 37% of patients had indirect signs of pulmonary hypertension on echocardiogram prior to NICU discharge. A patent ductus arteriosus was present in 57% of patients; of those, 37% required surgical correction. BPD was the only comorbidity significantly different among groups (UC: 21%, CC: 65% P=0.01). The rest of the patient characteristics and comorbidities didn’t differ between groups (Tables **2** and **3**), respectively. Overall, during the duration of the study, 48% of all infants were liberated from mechanical ventilation and 37% were successfully decannulated.

### 3.2 Significantly Lower Mortality was Evident in the Comprehensive Care Group

**Fig. (2):**
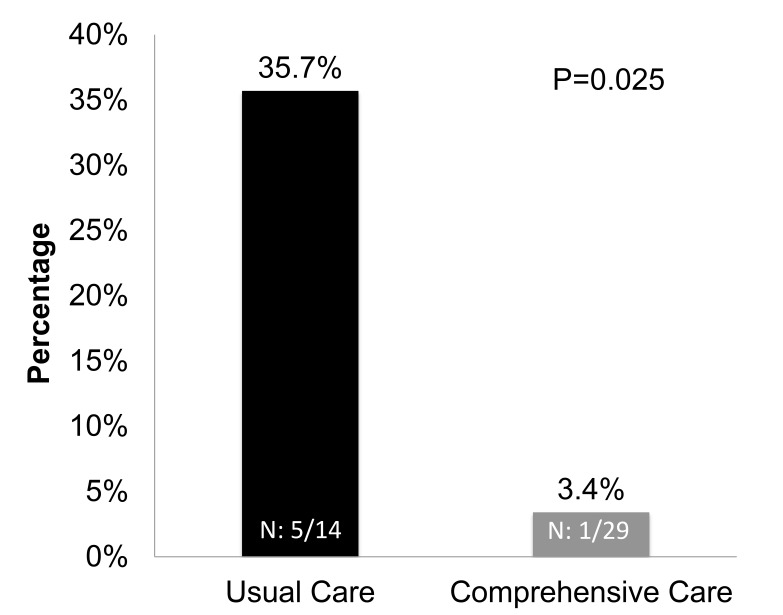
Comprehensive care intervention decreased mortality in technology-dependent NICU infants after discharge.

The total mortality rate was 13.9% (6/43). Analysis of mortality between groups was significantly lower in CC (3.4%, 1/29) compared to UC (35.7%, 5/14), RR, 0.09 [95%CI, 0.12-0.75], P=0.025. The average age at death was 17.9 months: 13.8 months in the CC versus 18.7 months in UC, which was significantly lower in the CC group. After adjustment for gestational age (<28 weeks; ≥28 weeks), RR for mortality was 0.11 [95% CI, 0.013-0.96], P=0.046, number needed to treat (NNT): 3.1. (Fig. **2**). After adjusting for BPD in the regression model evaluating the association between the intervention and mortality, the RR for intervention was 0.13 (95% CI, .01-1.2; P=0.076). Causes of death on UC included: three related to cardiorespiratory arrest after decannulation, septic shock, and acute hypoxemia with bradycardia leading to cardiorespiratory failure. The cause of death on CC was secondary to pulmonary hypertension and persistent hypoxemia.

Significantly lower mortality (1/29, 3.4%) in the comprehensive care group as compared to usual care (5/14, 35.7%); RR, 0.09 [95%CI, 0.12-0.75], P=0.025.

### 3.3 Comprehensive Care Reduced Total Hospital Admissions

There were a total of 127 admissions over a period of 5-½ years in our cohort; 65 admissions reported in CC versus 62 in UC. CC reduced total hospital admissions to 78 per 100 child-years versus 162 for UC; RR, 0.48 [95% CI, 0.25-0.93], P=0.03. After adjustment for gestational age (<28 weeks), the total number of admissions was significantly lower in the CC group; RR, 0.50 [95% CI, 0.25-1.00, P=0.05].

### 3.4 Rate of Readmission in the First 30-days after NICU Discharge was Comparable Among Groups

Among our cohort, the mean number of days to first admission post NICU discharge was 84 days. The 30-day readmission rate for CC was 21% compared to 36% in UC; RR, 0.58 [95% CI, 0.21-1.58], P=0.29). No significant differences between groups were noted in number of days before first readmission after adjustment for gestational age (<28 weeks), RR, 0.48 [95% CI, 0.12-1.88], P=0.29.

### 3.5 Mechanical Ventilation Removal in Comprehensive Care is Higher than Usual Care

**Fig. (3):**
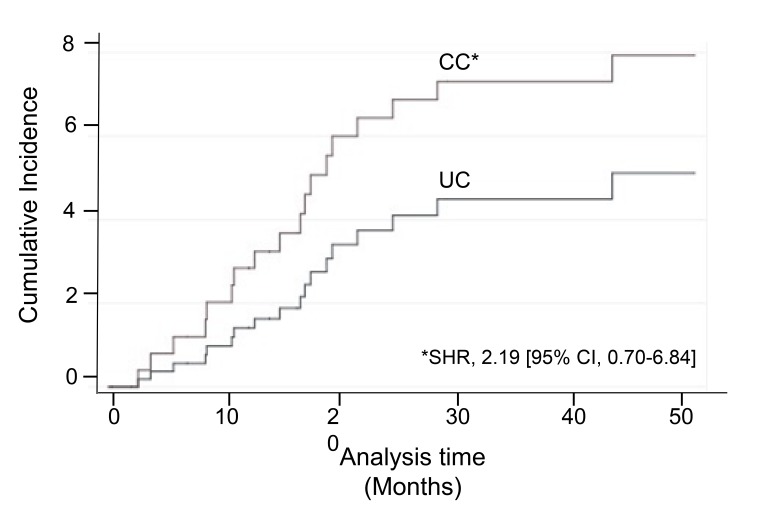
Competing-risk regression (treating death as a competing risk). Hazard of having mechanical ventilation removal in CC was two times higher than UC. CC: Comprehensive Care, UC: Usual Care.

Of the 86% (37/43) of infants who required mechanical ventilation at NICU discharge, successful liberation occurred in 56% (14/25) and 33% (4/12) of infants in the CC and UC, respectively. Median age at mechanical ventilation liberation was 24 months for CC and 22 months for UC group. Analysis by groups showed that the hazard of having mechanical ventilation removal in CC was two times higher than UC during the study time; SHR, 2.19 [95% CI, 0.70-6.84] (Fig. **3**).

### 3.6 Time-to-Decannulation was not Statistical Significant between Groups

Decannulation occurred in 38% (11/29) of infants in the CC versus 35% (5/14) in UC. Median age at decannulation was 32 months and 29 months for the CC and UC, respectively. There was no statistical difference in time-to-decannulation between groups; treating death as a competing-risk; SRR, 1.09 [95% CI, 0.37-3.15]. Data summary presented in (Table **4**).

## 4 DISCUSSION

This is a single site retrospective study that compared the health care outcomes of forty-three (N=43) technology-dependent infants discharged from NICU with a tracheostomy receiving CC in an enhanced medical home or UC. Previous studies have shown that CC reduces hospitalizations in child with medical complexity and prematurity [11, 13]. While the infants studied had a diversity of diagnoses that required tracheostomies including BPD, TEF and CDH, our data suggests CC could successfully reduce the rate of mortality rate, total number of hospital admissions per 100-child years and increase the hazard of having mechanical ventilation removal at 5 years. The mortality rate in our cohort was 13.9%, slightly lower than previous studies that explored the mortality associated with the presence of a mechanical ventilator and tracheostomy in children [14-21] (Table **5**). Our data shows that CC as an intervention decreased mortality in technology-dependent NICU infants after discharge. Although in our cohort, CC had an increased amount of patient with BPD and a lower median birth weight, regression model adjustment demonstrated similar trends for mortality toward intervention.

To the best of our knowledge, no previous studies have documented a reduction in mortality in technology-dependent infants discharged form the NICU following a CC intervention. The significant reductions in morbidity and mortality provided by CC can be partially explained by the availability and accessibility of experienced trained primary care providers and pediatric pulmonologists in a multidisciplinary medical home. The extent of care coordination and frequent interactions allow for a more proactive approach to healthcare, which allows for earlier treatments with reduced complications and improved health care outcomes.

Analysis of total number of admissions in our cohort was 127, meaning an average of 3 hospital admissions in 5 years per child, similar to a previous study with a 3.8% admission rate after tracheostomy during a 5-year period [4]. Several other studies have documented increased hospital readmissions of technology-dependent infants during the first year of life [22, 23]. We looked at both the total number of admission and the rate of readmission in the first 30-days after NICU discharge. The 30-days readmission rate was 15% less in CC (21% versus 36% in UC). This observation may be accounted for a more active role of providers in the HRCC. With providers available both in clinic during the day and via phone at night, this may have encouraged earlier intervention to seek medical advice and avoid the necessity of higher level of care. Although, the 30-day readmission rate was not significantly different between groups, earlier CC intervention may be important to avoid mortality in this high-risk population.

Prior studies have demonstrated that children who cannot be able to wean off the respiratory support by age 5 and get decannulated by age 6, then it is unlikely to occur [16]. Rate of mechanical liberation and decannulation for usual care was similar as previous studies reported (Table **5**). Infants who were followed in the CC group had a two times higher hazard of having mechanical ventilation removed as compared with UC. This means that a CC approach can be an effective intervention in earlier achievement of a non-technology dependent state. After data analysis using death as a compete risk, our data showed no significant differences between comprehensive and usual care groups in time-to-decannulation. However, infants in our retrospective study were followed for just 5-½ years, the timeframe may not be sufficient to demonstrate a difference between groups.

Our study provides essential information to primary care providers to develop a health care plan in agreement with the families expectations in order to understand the potential complications and associated morbidity and mortality with long-term care of technology-dependent infants [24]. Due to the intrinsic nature of a retrospective study in a single site and the implications of the sample power of our cohort, several limitations could affect our observations and findings. We conducted an analysis using a retrospective chart review that focused on technology-dependent patient discharged from the NICU with a diagnosis of tracheostomy. Patients with other types of technology that require a multidisciplinary care approach were not included in this study. We did not evaluate for neurodevelopmental outcomes during the study but we excluded patients with severe HIE and DNR status. We also excluded patients with concurrent comorbidities that can prolong or prevent mechanical ventilation liberation and decannulation. Additionally, this was a single site study and different centers may have distinctive protocols for mechanical liberation and decannulation. Furthermore, the amount of care provided in CC may not be feasible in all healthcare settings. Although a detailed cost analysis for CC versus UC in this specific cohort was not the aim of the study, an estimation of the potential cost may be estimated based on our previous study [11]. In this publication, we established that the estimated total mean cost per child-year total payment in the UC was $25,726 versus $14,467 in CC. A total cost reduction of $11,259 per child-year was evident and statistically significant (P=0.02). A reduction in hospital care was significantly lower in the CC group ($9,343) versus UC ($24,213), P=0.001. In our cohort CC reduced total hospital admissions to 78 per 100 child-years versus 162 for UC; RR, 0.48 [95% CI, 0.25-0.93], P=0.03. Additional studies with larger cohort may be necessary to evaluate for other significant outcomes or additional potential health care cost reductions.

## CONCLUSION

Few pediatric interventions decrease mortality and improve NICU outcomes. Our study explores the effect of comprehensive medical care as an intervention to improve the outcomes of tracheostomy-dependent NICU graduates. The data suggests a significant decrease in mortality, total number of hospital admissions and increase in the hazard of having mechanical ventilation removed following a comprehensive medical care versus usual care. Additional multicenter studies comparing comprehensive care versus usual care are needed.

## Figures and Tables

**Table 1:** Comprehensive care offers an enhanced-medical home model that includes a family-center approach to promote prompt effective care with the goal to reduce serious illnesses.

**Intervention**	**Usual Care **	**Comprehensive Care**
Experienced caregivers knowledgeable about each patient and available 24/7 by cell phone with EMR access	No	Yes
High priority given to prevent unnecessary ED visits and hospitalizations	No	Yes
Identification each day of all hospitalizations and ED visits to assure prompt follow up and care coordination	No	Yes
Same day and walk-in appointment	No	Yes
Subspecialist in the clinic	No	Yes*
Bilingual providers (Spanish, English)	Sometimes	Always
Coordination of care by Nurse Practitioners	No	Yes
Nutritionist and Social worker in clinic	No	Yes
Low provider-to-patient ratio	No	Yes#

*Pediatric Pulmonary as a Primary care provider, Adolescence medicine, Neurology, Gastroenterology, Allergy and Immunology, Infectious diseases in clinic one per month and on call 24/7 over phone. ^#^Provider-to-patient ratio: (<1:50).

**Table 2:** Baseline characteristics and demographics of technology-dependent BPD infants discharged from the NICU with tracheostomy were similar in both groups.

**Characteristics **	**Usual Care, N=14**	**Comprehensive Care, N=29**	**P value**
Female, N(%)	8(57)	10(34)	0.36
Race, N(%)	–	–	–
Caucasian	3(21)	4(14)	0.22
African American	7(50)	9(33)	–
Asian	1(7)	1(4)	–
Hispanics	3(21)	13(48)	–
Birth weight, grams median	2556	1548	0.2
Gestational age, wk, median	35	30	0.23
Health Insurance, N(%)	–	–	–
Medicaid	13(92)	20(69)	0.41
Age at NICU discharge, months	5.8	6.5	0.55
Age of tracheostomy, months	3	4.3	0.14

N(%): number and percentage of subjects; wk: weeks. P value <0.05 was considered statistically significant.

**Table 3:** Associated comorbidity of technology-dependent infants discharged from the NICU with tracheostomy.

**Morbidity, N(%)**	**UC, N=14**	**CC, N=29**	**Total, N=43**	**P value**
Bronchopulmonary Dysplasia	3(21)	19(65)	22(51)	0.01
Mechanical ventilation dependent	12(85)	25(86)	37(86)	0.97
SIMV-PC	8(57)	10(34)	18(41)	0.12
SIMV-VC	1(7)	4(13)	5(11)	0.49
PEEP+PS	1(7)	3(10)	4(9)	0.71
CPAP	0(0)	3(10)	3(7)	0.21
Oxygen dependent	6(42)	11(37)	17(40)	0.99
Gastrostomy	9(64)	21(72)	30(69)	0.26
Gastrojejunal tube	1(7)	0(0)	1(2)	0.24
Nissen fundoplication	2(6)	4(13)	6(20)	0.86
Intraventricular Hemorrhage	4(28)	2(7)	6(14)	0.13
Pulmonary Hypertension by ECHO*	4(28)	9(20)	13(30)	0.88
Indirect signs on ECHO	5(35)	11(78)	16(37)	0.74
Patent Ductus Arteriosus	8(28)	16(55)	24(57)	0.89
Surgical ligation	4(44)	5(31)	9(37)	0.39
Congenital Diaphragmatic Hernia	1(7)	1(3)	2(5)	0.75
Trachesophageal Fistula	1(7)	3(10)	4(9)	0.56
Oromaxilofacial Abnormalities	3(21)	4(13)	7(16)	0.82
Spinal Muscular Atrophy	1(7)	0(0)	1(2)	0.21
Trisomy 21	4(28)	3(10)	7(16)	0.28

N(%): number and percentage of subjects, UC: Usual Care, CC: Comprehensive Care. P value <0.05 was considered statistically significant. *Right ventricle systolic pressure [RVSP] above half systemic pressure, Echocardiogram (ECHO).

**Table 4:** Statistics Summary: Analysis of the outcomes of infants with tracheostomy discharged from NICU following usual care versus comprehensive care.

**Variable **	**UC**	**CC**	**Statistical Analysis**
Mortality, N(%)	5(35.7)	1(3.4)	RR, 0.09 [95%CI, 0.12-0.75], P=0.025
–	–	–	RR, 0.11 [95% CI, 0.013-0.96], P=0.046*
–	–	–	RR, 0.13 [95% CI, 0.014-1.24], P=0.076**
Total admissions#	162	78	RR, 0.48 [95% CI, 0.25-0.93], P=0.03
–	–	–	RR, 0.50 [95% CI, 0.25-1.00], P=0.05*
30-days readmission rate^ (%)	36	21	RR, 0.58 [95% CI, 0.21-1.58], P=0.29
–	–	–	RR, 0.48 [95% CI, 0.12-1.88], P=0.29*
MV liberation, N(%)	4(33)	14(56)	SHR, 2.19 [95% CI, 0.70-6.84]
Time-to-MV liberation (mo)	22	24	–
Decannulation, N(%)	5(38)	11(45)	SHR, 1.09 [95% CI, 0.37-3.15]
Time-to-decannulation, (mo)	29	32	–

*Adjusted for gestational age (<28 weeks). **Adjusted for BPD. SHR: Subhazard Ratio calculated using the competing-risk regression model. ^#^Number of total admissions per 100-child years. ^Rate of readmission in the first 30-days after NICU discharge.

**Table 5:** Published studies evaluating outpatient outcomes of technology-dependent children. Overall, the mortality rate in our cohort was 14%, slightly lower than previous studies that explored the mortality associated with the presence of a mechanical ventilator and tracheostomy in children.

**Follow Up **	**N **	**Mortality **	**MV Liberation**	**Decannulation**	**Reference**
**(Years)**	**-**	**N(%)**	**N(%)**	**N(%)**	**(Author, Year)**
12	56	2(4)	-	22(39)	Chen, 2017
15	144	6(4)	76(52)	-	McDougall, 2013
27	102	19(19)	69(67)	60(58)	Cristea, 2013
12	21	11(52)	14(66)	3(14)	Challapudi, 2013
5	30	8(27)	20(25)	5(17)	Dursun, 2010
5	47	47(21)	41(18)	-	Edwards, 2010
9	77	13(17)	17(22)	-	Gowans, 2007
5	11	4(36)	2(18)	-	Oktem, 2008
11.3	61	14 (23)	34(56)	23(38)	Total*
5.5	14	5(35)	4(33)	5(35)	UC**
5.5	29	1(3)	14(56)	11(38)	CC**
5.5	43	6(14)	18(48)	16(37)	Complete cohort**

UC: Usual Care, CC: Comprehensive Care . * Mean values between all previous studies (2008-2013). ** Summary of our data (2011-2017).

## LIST OF ABBREVIATIONS

BPD: = Bronchopulmonary Dysplasia
CC: = Comprehensive Care
CCHS: = Central Hypoventilation Syndrome
CDH: = Congenital Diaphragmatic Hernia
DNR: = Do Not Resuscitate status
ED: = Emergency Department
HIE: = Hypoxic-Ischemic-Encephalopathy
HRCC: = High-Risk Children’s Clinic
IVH: = Intraventricular hemorrhage
NICU: = Neonatal intensive care
ORL: = pediatric otorhinolaryngologist subspecialist
SIMV: = Synchronized Intermittent Mandatory Ventilation
TEF: = Tracheoesophageal Fistula
UC: = Usual Care
UTH: = The University of Texas Health Science Center at Houston
